# Renal cancer biomarkers: the promise of personalized care

**DOI:** 10.1186/1741-7015-10-112

**Published:** 2012-09-27

**Authors:** Naveen S Vasudev, Peter J Selby, Rosamonde E Banks

**Affiliations:** 1 The Institute of Cancer Research, Fulham Road, London SW3 6JB, UK; 2 Cancer Research UK Centre, Leeds Institute of Molecular Medicine, St James's University Hospital, Leeds, UK

**Keywords:** Biomarkers, epigenomics, genomics, renal cell carcinoma, transcriptomics

## Abstract

Significant advances in our understanding of the biology of renal cell carcinoma (RCC) have been achieved in recent years. These insights have led to the introduction of novel targeted therapies, revolutionising the management of patients with advanced disease. Nevertheless, there are still no biomarkers in routine clinical use in RCC. Tools used routinely to determine prognosis have not changed over the past decade; classification remains largely morphology based; and patients continue to be exposed to potentially toxic therapy with no indication of the likelihood of response. Thus the need for biomarkers in RCC is urgent. Here, we focus on recent advances in our understanding of the genetics and epigenetics of RCC, and the potential for such knowledge to provide novel markers and therapeutic targets. We highlight on-going research that is likely to deliver further candidate markers as well as generating large, well-annotated sample banks that will facilitate future studies. It is imperative that promising candidates are validated using these resources, and in subsequent prospective clinical trials, so that future biomarkers may be used in the clinic to personalize patient care.

## Renal cell carcinoma

Renal cancer is the eighth most common cancer in the UK. There are over 270,000 new cases worldwide each year, 9,000 of which occur in the UK [1,2]. It accounts for more than 100,000 deaths across the world per annum [2]. The vast majority (approximately 90%) of renal cancers arise in the renal parenchyma and are termed renal cell carcinomas (RCC). The incidence of RCC has been steadily rising over the past 20 years in many countries and this is thought to be only partially explained by the increased rate of incidental diagnosis.

The most common histological subtype of RCC is the conventional or clear cell (ccRCC) type accounting for 70% to 80% of cases. Central to the biology of ccRCCs, which form the focus of this review, is loss of function of the *Von Hippel-Lindau (VHL*) tumour suppressor gene (TSG), located on chromosome 3p. More than 90% of sporadic ccRCCs have *VHL *involvement, almost defining this subgroup of tumours [3,4]. Loss of function of the VHL protein leads to stabilisation of hypoxia-inducible factors, nuclear transcription factors that in turn can activate the transcription of many genes including those encoding vascular endothelial growth factor (VEGF) and platelet derived growth factor.

The majority (60% to 70%) of patients present with localised disease, for which radical or partial nephrectomy remains the standard of care and is largely curative. However, approximately one third of these patients will subsequently relapse and die of their disease. Accurately determining the risk of relapse after nephrectomy is a key issue for patients and clinicians. This would not only inform and personalize imaging and follow-up schedules but also determine the risk:benefit ratio for adjuvant therapy, if on-going trials are positive. Current nomograms used to determine risk are still based on clinicopathological criteria only, and were developed more than a decade ago [5]. Such scoring systems are reasonably accurate at a population level but distinguishing risk for individuals, particularly those deemed at intermediate risk, remains poor. This is a key area where biomarkers are urgently required in RCC.

**Figure 1:**
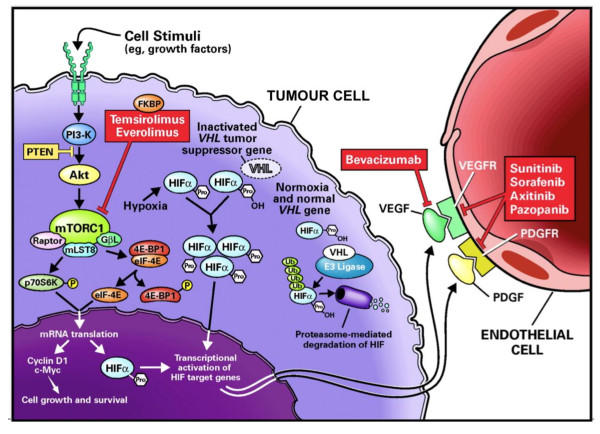
**Biological pathways targeted for therapy in renal cell carcinoma based on a knowledge of the underlying genetic changes and downstream biological consequences**. Loss of function of the VHL tumour suppressor gene leads to stabilisation of hypoxia-inducible factor alpha (HIFα). Activated HIF translocates into the nucleus and leads to the transcription of a large number of hypoxia-inducible genes including vascular endothelial growth factor (VEGF) and platelet derived growth factor (PDGF). Mammalian target of rapamycin (mTOR) is a kinase within the PI3K/Akt pathway that can promote cell growth and survival pathways as well as causing accumulation of HIF. Bevacizumab is a monoclonal antibody to VEGF whilst sunitinib, sorafenib, axitinib and pazopanib are VEGF receptor tyrosine kinase inhibitors. These agents are thought to primarily function as antiangiogenic agents, inhibiting ligand binding or downstream receptor signalling of VEGF and PDGF on endothelial cells. Temsirolimus and everolimus inhibit the kinase activity of the mTOR complex 1 (mTORC1). Reproduced with permission from Elsevier©. From [66]. HIF: hypoxia-inducible factor; mTOR: mammalian target of rapamycin; mTORC1: mTOR complex 1; PDGF: platelet derived growth factor; PTEN: phosphatase and tensin homolog; VEGF: vascular endothelial growth factor; VEGFR: vascular endothelial growth factor receptor.

Insights into the biology of ccRCC have directly led to the recent introduction of a number of effective systemic therapies (see Figure 1). Antiangiogenic VEGF receptor (VEGFR)-targeted tyrosine kinase inhibitors (TKI), such as sunitinib and pazopanib, are established as front-line therapy for patients with advanced RCC. However, the clinical benefit an individual patient will derive from such therapy is highly variable and largely unpredictable. Between 20% and 30% of patients with ccRCC derive no benefit from first-line TKI treatment [6,7]. In addition, these drugs are toxic and expensive. Modern medical practice demands value for money. There is thus great impetus to discover biomarkers in RCC that can identify the subpopulation of patients destined to gain maximal benefit from any given drug. Numerous studies, variably examining clinicopathological criteria, VHL status, serum cytokines and angiogenic factors in relation to TKI response have been published, and reviewed elsewhere [8].

Despite the tremendous spectrum of behaviour that characterises ccRCCs, the current approach to the management of patients with these tumours remains largely generic. Robust and clinically validated biomarkers are required if the long-held promise of personalized medicine is to be realised. In this review, we summarise some of the most recent and promising areas of genetic and epigenetic biomarker research in ccRCC. We also highlight a number of large biomarker initiatives in RCC that are underway and, finally, we discuss some of the issues related to successfully bringing RCC biomarkers into the clinic. A detailed review of protein biomarkers of RCC and the potential of proteomics strategies in this area is beyond the scope of the current review and has been covered elsewhere by the authors [9]. In addition, although the current focus is on ccRCC, much greater research is also urgently required into the other, less common, subtypes of RCC, to define the biology of these tumours and lead to rational therapeutic design.

## Recent advances in genetic, epigenetic and transcriptomic understanding of clear cell renal cell carcinoma

Tremendous progress has been made in recent years in terms of our understanding of the genetic basis of cancer. In particular, the advent of second-generation DNA sequencing technology is allowing researchers to start to systematically catalogue the thousands of somatic mutations that can typically be found in adult cancers and it is expected that tens of thousands of cancer genomes will be sequenced in the next 5 years [10]. This is, therefore, a time of great anticipation and it is expected that studies at genetic, epigenetic and transcriptomic levels will together identify the full complement of key driver mutations and epigenetic contributions across all cancer types, including RCC [11]. Complemented by information obtained at the level of the proteome [12], it is expected that these studies will together ultimately result in the identification of novel biomarkers and therapeutic targets of cancer.

### DNA

Amongst the most notable genetic abnormalities associated with ccRCCs are loss of chromosome 3p (70% to 80%) and gain of chromosome 5q (50% to 60%) [13]. Loss-of-function mutations in the remaining VHL allele are thought to represent an early event in ccRCC development but are not sufficient alone to drive tumour growth. A second major TSG recently implicated in ccRCC is the SW1/SNF chromatin remodelling complex gene *polybromo1 *(*PBRM1*), with truncating mutations found in 41% of the 227 cases tested [14]. Other genes, such as *SET domain containing protein 2 *and *Jumonji AT-rich interactive domain 1C *have also been implicated, although at much lower frequency (3%) [15]. More recently, mutations in *BRCA related protein-1 *(*BAP1*) have been reported, with inactivation of BAP1 protein in 15% of ccRCCs. Interestingly, mutations in *PBRM1 *and *BAP1 *were largely observed to occur exclusively, suggesting that simultaneous loss may be disadvantageous to the tumour [16]. In comparison with the *PBRM1 *mutation, BAP1-deficient tumours were of higher grade and had distinct gene expression profiles. Distinguishing these genetically distinct subgroups may, therefore, have important prognostic and therapeutic implications for individual patients. It is of note that this may be achievable at the protein level, examining BAP1 protein expression by immunohistochemistry, for example, which is less expensive, has a higher throughput and is more routinely available [16].

The real message from these studies is perhaps that even relatively large-scale analyses may be underpowered to capture the full spectrum of mutations that drive these tumours and must be differentiated from the many bystanders. There is, therefore, a need for even larger studies, ultimately including thousands, rather than hundreds, of samples and covering whole genomes so that potentially important non-exomic changes can also be identified. Such studies are in fact underway (see later) and are required if further significant insights, beyond *VHL*, *PBRM1 and BAP1*, are to be gained.

### Gene expression microarrays

Gene expression microarrays represent a promising method by which to subclassify tumours, both across subtypes and within clear cell carcinomas. Importantly, of course, they encompass both upstream genetic and epigenetic changes. Such approaches may also provide prognostic information that can be implemented in daily clinical practice. A proof of concept has been established in other tumour types, such as breast cancer: MammaPrint is a Food and Drug Administration approved 70-gene signature, stratifying tumours as high or low risk, which has been widely adopted into clinical practice in countries such as the USA. Studies to date in ccRCC have established that these tumours can also be stratified based on gene expression profiling, and that this may provide information independent of stage and grade [17-20]. However, these studies have typically been small, with limited numbers of genes analysed, and not independently validated. Indeed, in a recently published meta-analysis of gene-expression studies in ccRCC, just six studies were included after excluding those with fewer than 20 tumours, those with fewer than 5,000 genes analysed, those containing no clinical data and redundant publications of previous data [21]. The meta-array assembled gene expression data from 480 tumours, encompassing 6,386 genes. Based on previous work by the same authors, the study demonstrated the ability of such profiling to segregate ccRCCs into two distinct subtypes, termed ccA and ccB. ccA tumours relatively overexpressed genes associated with hypoxia, angiogenesis and fatty acid metabolism and carried a favourable prognosis in comparison with ccB tumours, which overexpressed a more aggressive panel of genes associated with epithelial-mesenchymal transition, cell cycle and wound healing. Interestingly, rates of VHL involvement were similar between the two groups [18]. A third, small (14%) cluster of tumours could also be identified, 82% of which were classed as VHL wild-type. Importantly, a histological review of these cases demonstrated that over half displayed deviations from classical clear cell features, suggesting that such tumours may warrant distinct classification.

Another recent study combined copy number analysis with gene expression analysis to identify potential novel subtypes and therapeutic targets in ccRCC. The study examined 54 cases of sporadic ccRCC, and found 350 concordantly gained and overexpressed genes. Gain in chromosome 5q was observed in 30% of cases, and *stanniocalcin *(*STC2*) and *versican *(*VCAN*) were identified as potential oncogenes in ccRCCs, which appear to act by inhibiting cell death [22]. The study is notable for describing gain-of-function aberrations, rather than the more commonly described loss of function in tumour suppressors; these gain-of-function aberrations may represent more direct therapeutic targets

Histopathological classification of RCC can prove challenging in some cases [23,24]. The ability to differentiate between subtypes is important, because prognosis and treatment can vary, and there are implications for clinical trial recruitment. Gene expression signatures, using as few as 10 genes, have shown more than 90% accuracy in distinguishing between clear cell, papillary and chromophobe RCCs as well as benign oncocytomas [25]. Such profiling may prove to be of value in the clinic if shown to have the ability to subtype currently unclassifiable tumours, or in cases that are otherwise difficult to distinguish (for example, eosinophilic tumours). In addition, the increasing interest in neoadjuvant therapy means that pathologists are required to make initial diagnoses on much more limited amounts of tissue, derived from core biopsies alone, where expression profiling may also prove useful.

### Single nucleotide polymorphisms

Large genome-wide association studies have recently reported SNPs that may increase the risk of an individual developing RCC [26,27]. Such genetic variation within our germline, in addition to somatic mutations within tumours, may also help to explain observed differences in response and toxicity to anticancer agents.

A number of SNP-based studies in RCC addressing the response to TKI therapy have recently been published. In the largest study of 397 patients treated with pazopanib, 27 polymorphisms amongst 13 genes, including those related to angiogenesis (*VEGFA*/*IL-8*/f*ibroblast growth factor 2*), metabolism (*cytochrome P_450 _*(CYP) 3A4*/5*)*}*) and transport (*ATP-binding cassette *(ABC) B1) were evaluated. Two IL-8 polymorphisms, linked to increased gene expression, were associated with a significantly shorter median PFS (27 weeks) than those carrying the wild-type genotype (48 weeks) (*P *= 0.01) [28]. It is of note that IL-8 has recently been identified as a potential driver of resistance to TKIs [29], making the results of biological relevance. A second study, conducted prospectively, examined both response (n = 89) and toxicity (n = 95) to sunitinib in patients with ccRCC. A total of 16 polymorphisms were examined in nine genes. Two *VEGFR3 *missense polymorphisms were associated with reduced PFS and a high metabolising variant of CYP3A5*1 was associated with increased toxicity on multivariable analysis. However, the reported SNPs involving IL-8 were not demonstrated in this study [30]. In a retrospective study of 136 patients with metastatic ccRCC treated with sunitinib, 30 SNPs in 11 genes were examined, and correlated with PFS. Survival was significantly improved in relation to SNPs in *CYP3A5*, ligand-activated nuclear receptor *NR1I3 *and *ABCB1*, but not in *VEGFR3 *[31].

Thus, studies to date in this area show little concordance. The frequency of the reported SNPs in the tested populations was typically low, highlighting the need for such studies to be much larger to increase their power to detect significant differences. Furthermore, the applicability of the results to populations of differing ethnicity is also unknown.

### DNA methylation

DNA methylation represents the best characterised mechanism by which cancer cells can epigenetically regulate gene expression. Methylation of cytosine residues within CpG dinucleotides can alter the transcription rate of a given gene and bring about transcriptional silencing. Cancer cells often demonstrate TSG inactivation as a result of aberrant promoter hypermethylation [32].

In sporadic ccRCC, the VHL TSG is inactivated via methylation in approximately 10% to 30% of cases [3,4,33]. Methylation studies, including recent genome-wide based approaches, have now identified a large number of other candidate TSGs inactivated by hypermethylation in ccRCC, which, in many cases, occur at high frequency within the studied sample sets [34,35]. The Ras association domain family 1 gene, for example, codes for a protein that functions as a negative regulator of the cell cycle and is methylated in approximately 45% of cases [33]. Secreted frizzled related protein 1, which antagonises Wnt signalling, is methylated in 34% to 68% of ccRCC tumours [36,37]. Many other examples exist, as recently reviewed [38].

Such studies clearly provide further insights into the biology of ccRCC but can markers of methylation also serve as novel biomarkers in the clinic? Correlations between methylation status and patient outcome have been reported although none have been validated. Methylation of gremlin1, a protein that antagonises growth factor signalling, has been correlated with a poorer overall survival in patients with ccRCC. The study included 185 patients, 40% of whom had methylation of the gremlin1 gene [39]. Methylation of GATA-binding protein 5 has recently been correlated with the development of metastasis (*P *= 0.005) and decreased progression-free survival (*P *= 0.005, hazard ratio = 4.59) in 84 patients with ccRCC [40]. In another study of 69 patients with ccRCC, in the 19% of patients with methylation of signal peptide CUB EGF-like domain-containing protein 3, risk of cancer death or relapse was significantly increased (*P *= 0.0046) [35].

Intriguing data have recently been published from studies in bladder cancer, another urological malignancy, suggesting that urinary methylation markers may be used to diagnose bladder cancer [41]. Furthermore, such markers may also predict the progression of early bladder lesions to muscle-invasive tumours with a high degree of accuracy [42]. It is also possible that urinary DNA methylation markers may be used in RCC to allow early detection of disease, potentially using a pan-urological panel of markers [43].

### microRNA

miRNAs are single-stranded, non-coding RNAs approximately 22 nucleotides long that are emerging as a potentially important and novel source of epigenetic biomarkers in RCC. miRNAs function by regulating gene expression at a post-transcriptional level, binding to target mRNA, typically resulting in translational downregulation, inhibition and/or mRNA degradation but also, more rarely, upregulation. miRNAs are altered in many cancers, including RCC, and can affect many tumourigenic pathways including cell cycle regulation, proliferation, cell motility, metastasis, apoptosis and angiogenesis.

Many alterations in the expression of miRNAs in RCC have been described to date (for reviews see [44,45]) and provide fresh insight into the aetiology and biology of these tumours. miR210, for example, has consistently been reported to be upregulated in ccRCC in response to hypoxia, and promotes anaerobic respiration and cell cycle progression and inhibits pro-apoptotic signalling [46-48]. Furthermore, expression of miR210 has been correlated with a significantly poorer overall survival (*P *= 0.0006), even among a small number of patients (n = 31) [49].

miRNA-based signatures may also allow for improved classification of tumour subtypes. In a recent study of 94 fresh frozen samples, composed of normal renal epithelium and clear cell, papillary and chromophobe RCC subtypes as well as oncocytomas, 91 miRNAs were found to be significantly differentially expressed. Clear cell tumours were shown to be more closely related to papillary RCC and both were distinct from chromophobe and oncocytomas, which were more closely related. Tumours could be classified using unique miRNA signatures in a maximum of four steps. The system had a sensitivity of 97% in distinguishing normal from RCC, 100% for clear cell RCC, 97% for papillary subtype and 100% accuracy in distinguishing oncocytoma from chromophobe tumours. This latter distinction is notoriously difficult based on morphology alone [50].

It is worth noting that miRNAs are stable and can in fact be reliably measured in formalin-fixed paraffin-embedded material. Furthermore, as recent studies have shown, miRNAs can also be measured in serum and serve as potential diagnostic markers of disease [51,52].

Finally, and perhaps most exciting, miRNAs may serve as novel therapeutic targets. Because any given miRNA may target hundreds to thousands of genes, such an approach may have the capacity to 'hit' several pathways simultaneously. However, at present, such an approach remains in its infancy across all cancer types, not just in RCC.

## Current renal cell carcinoma biomarker initiatives

A number of large-scale biomarker initiatives are underway in RCC, some of which are described below.

### CAGEKID

**Figure 2:**
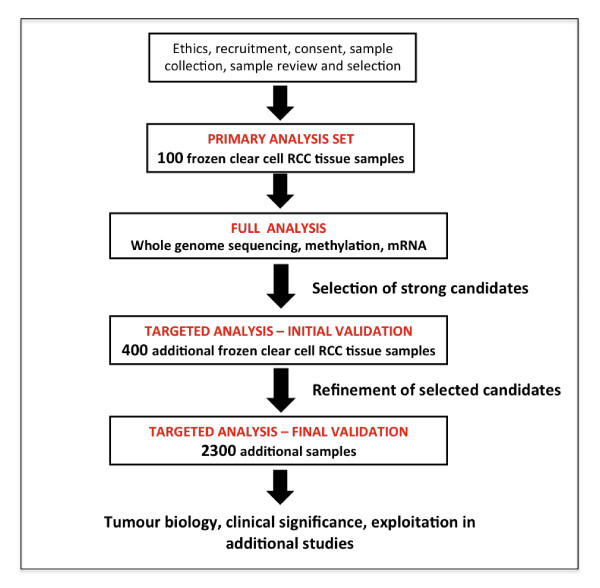
**Cancer Genomics of the Kidney (CAGEKID) study schema**. RCC: renal cell carcinoma.

The Cancer Genomics of the Kidney (CAGEKID) consortium [53], funded by the European Union (EU) (total €10 million), aims to carry out comprehensive genetic, epigenetic and transcriptomic analysis in ccRCC. Extensive characterisation of 100 patients and two phases of targeted validation in a further 400 and 2,300 patients will be performed. The study forms part of the International Cancer Genome Consortium initiative [11] and, as such, all samples entered are subject to pathology panel review and minimum standards used in terms of viable tumour cell number. The consortium is composed of 14 partners from 6 EU countries (plus Russia), including the Czech Republic. A schema for the CAGEKID study is shown in Figure 2. The study is currently in its initial validation phase.

### Evaluation of biomarkers in patients with renal cell carcinoma

As part of a National Institute for Health Research funded programme ('Biomarker pipeline'), tissue and fluid samples from patients with RCC are being collected at 10 UK centres, with a target of 600 patients at baseline, longitudinal sampling in a further 200 patients and long-term follow-up in all. Samples are being collected according to strict standard operating procedures, together with clinical data via case report forms. An important remit of the study is the careful evaluation of existing and future putative prognostic and longitudinal monitoring protein biomarkers for use in the clinic, incorporating validation of developed and existing assays. The sample bank will also be available for future gene and protein studies [54].

### EuroTARGET

EuroTARGET (TArgeted therapy in Renal cell cancer: GEnetic and Tumour related biomarkers for response and toxicity) is another European collaboration, composed of 12 partner organisations from 8 countries, funded by the European Commission under the Seventh Framework Programme. The aim of the study is to identify predictive biomarkers of response and toxicity to targeted therapy in patients with RCC using approaches that include germline genome analysis and tumour gene expression and methylation studies [55].

### SCOTRRCC

The Scottish Collaboration on Translational Research into RCC (SCOTRRCC) is a Scotland-wide initiative involving 10 centres, aimed at banking clinical samples from newly diagnosed patients with RCC to address various clinical and scientific research questions. Samples will be collected, processed and stored in a robust and uniform manner, accompanied by comprehensive clinical annotation, providing a further highly valuable biobank.

### PREDICT Consortium

personalized RNA Interference to Enhance the Delivery of Individualised Cytotoxic and Targeted therapeutics (PREDICT) is a European collaboration aimed at identifying predictive biomarkers of response to sunitinib and everolimus in patients with RCC [56]. Patients within the study provide consent to receive neoadjuvant therapy, allowing the collection and comparison of tissue both before and after exposure to the drug. Tumours will be comprehensively genomically profiled and high-throughput screens using short hairpin RNA and small interfering RNA will be used to identify and validate functionally important genomic or transcriptomic predictive biomarkers of individual drug response in patients.

### TCGA

The Tumour Cancer Genome Atlas (TCGA) is a US initiative funded by the National Institutes of Health that sets out to comprehensively genomically profile 20 different tumour types, including both ccRCC and papillary RCC. The study has already banked its target of 500 ccRCC samples, and aims to conduct whole genome sequencing in 50 of these cases. As with other initiatives, the data will be made available to the scientific community [57].

## The future of using biomarkers for personalized medicine for renal cancer

Treatment options and outlook for patients with renal cancer have seen significant improvement in recent years: surgical techniques have improved; tumour-ablative procedures are more widely available; and effective targeted agents have been discovered. But what next? Further major advances are likely to require the introduction of biomarkers into clinical practice to personalize patient care.

**Figure 3:**
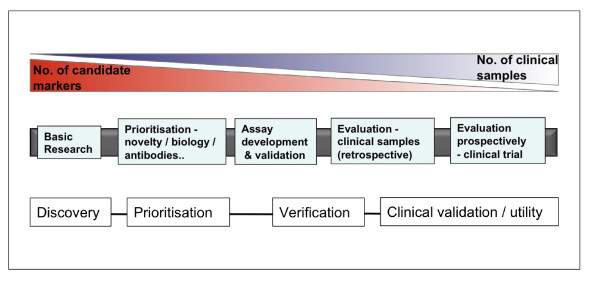
**Biomarker pipeline**. Biomarkers must be carefully evaluated along each phase of the pipeline for successful adoption into clinical practice.

An abundance of potential candidate RCC biomarkers exist in the literature, yet none have progressed beyond the discovery phase, an issue that has plagued biomarker research across all cancer types [58]. Some of the most promising markers in RCC are in fact proteins, such as B7-H1 and insulin-like growth factor II mRNA binding protein 3, that have been shown in RCC to have strong, independent prognostic ability, have been externally validated and add value to existing nomograms [59,60], yet appear to have stalled at this point in the 'biomarker pipeline' (Figure 3). Even C-reactive protein, a more routine and easily measured marker, has not been evaluated further, despite having been shown in multiple studies to be of prognostic value and occur not just as an inflammatory marker but be produced by RCC cells [61]. If genomic markers are not to similarly stall in their development, it is important that these issues are urgently addressed. As others have argued [62], to date, too much emphasis has been placed on the discovery phase of biomarker research and not enough on validation and integration of markers into clinical care. Thus, alongside current discovery initiatives, a priority in RCC biomarker research must be to validate, in or out, existing promising markers, following robust assay development.

Can biomarkers be successfully integrated into clinical practice in RCC? It is certainly appealing to envisage a future in which patient management is influenced by molecular information that can be reliably provided in 'real-time'. Thus, from a single renal biopsy, a wealth of information would be gleaned at both a genetic and protein level, defining the tumour more so on its molecular profile than its site of origin. Based on this notion, initiatives such as Cancer Research UK's Stratified Medicines Programme are already exploring how the National Health Service can provide molecular profiling routinely for all cancer types and lay the foundations for a national service that can deliver standardised, high quality, cost-effective genetic testing of tumours [63].

Among the many issues relating to the successful introduction of individualised cancer care, perhaps one the greatest challenges comes from the increasing recognition that individual tumours, across many cancer types, are themselves highly heterogeneous [64]. The remarkable degree of heterogeneity that exists within individual ccRCCs has recently been elegantly demonstrated [65]. Using multi-region genetic analysis, this study showed that the majority (approximately two thirds) of mutations are not present in every region of a tumour, and that a single biopsy would capture only a minority of the genetic aberrations present. Furthermore, different areas of the same tumour variably returned either a favourable or unfavourable prognostic profile, using the gene expression array described above [18], suggesting possible diversity amongst biologically relevant (driver) mutations.

Such heterogeneity of course is a feature of all cancers and potentially carries significant implications for successful biomarker validation and the delivery of personalized care [64]. A single biopsy may not be representative of the tumour as a whole and even multiple sampling post-nephrectomy may be inadequate. A further level of complexity is also added by the fact that the signature of the primary tumour may not necessarily reflect that of distant metastatic deposits [65]. What remains uncertain, however, is the extent to which these differences actually impact on the tumour phenotype and, for instance, the expression of protein biomarkers and therapeutic targets. For now, the key message is that heterogeneity exists and must be considered along the route to successful biomarker validation.

## Conclusions

This is a highly promising time in the field of RCC biomarker research. The advent of high-throughput molecular profiling technologies is leading to a revolution in our understanding of the biology and make-up of RCC and, in parallel, a number of large-scale, collaborative biomarker discovery initiatives are underway. It is expected that these studies, complemented by proteomic initiatives, will identify further novel candidate biomarkers of RCC. Equal efforts must then be applied to the clinical validation phase for such studies to be rendered worthwhile.

## Abbreviations

ABC: ATP-binding cassette; BAP1: BRCA related protein-1; CAGEKID: Cancer Genomics of the Kidney consortium; ccRCC: clear cell renal cell carcinoma; CYP: cytochrome P450; EU: European Union: IL-8: interleukin 8; mRNA: messenger RNA; miRNA: microRNA; PFS: progression-free survival; PBRM1: polybromo1; RCC: renal cell carcinoma; SNP: single nucleotide polymorphism; TKI: tyrosine kinase inhibitor; TSG: tumour suppressor gene; VEGF: vascular endothelial growth factor; VEGFR: vascular endothelial growth factor receptor; VHL: Von Hippel-Lindau.

## Competing interests

The authors declare that they have no competing interests.

## Authors' contributions

NV wrote the first draft of the manuscript, which RB and PS critically reviewed. All authors read and approved the final manuscript.

## Author information

NV is a Medical Oncologist and Research Fellow at the Institute of Cancer Research, Royal Marsden Hospital, London. PS is Professor of Cancer Medicine at the St James's Institute of Oncology, University of Leeds and Director of the Leeds Institute of Molecular Medicine (LIMM). RB is Professor of Biomedical Proteomics, LIMM. RB and PS lead the Clinical and Biomedical Proteomics Group, focused on biomarker and therapeutic target discovery in renal cell carcinoma. The authors are partners within both the EU CAGEKID and the National Institute for Health Research biomarker evaluation programmes.

## Pre-publication history

The pre-publication history for this paper can be accessed here:

http://www.biomedcentral.com/1741-7015/10/112/prepub
